# 1969. SARS-CoV-2 seroprevalence among patients with cancer and healthcare workers from an oncology referral center during the first year of COVID-19 vaccination in Mexico

**DOI:** 10.1093/ofid/ofac492.1594

**Published:** 2022-12-15

**Authors:** Rodrigo Villaseñor-Echavarri, Daniel De-la-Rosa-Martinez, Emmanuel Frias-Jimenez, Alexandra Martin-Onraet, Erika Ruiz-Garcia, Alonso Cruz-Cruz, Luis Alonso Herrera-Montalvo, Diana Vilar-Compte

**Affiliations:** Instituto Nacional de Cancerologia, Mexico City, Distrito Federal, Mexico; Instituto Nacional de Cancerologia, Mexico City, Distrito Federal, Mexico; Instituto Nacional de Medicina Genomica, Mexico City, Distrito Federal, Mexico; Instituto Nacional de Cancerologia, Mexico City, Distrito Federal, Mexico; Instituto Nacional de Cancerologia, Mexico City, Distrito Federal, Mexico; Instituto Nacional de Medicina Genomica, Mexico City, Distrito Federal, Mexico; Instituto Nacional de Medicina Genomica, Mexico City, Distrito Federal, Mexico; Instituto Nacional de Cancerología, Mexico City, Distrito Federal, Mexico

## Abstract

**Background:**

Cancer patients (CPs) with COVID-19 have an increased risk of adverse outcomes. In addition, CPs seem to have a lower immune response to SARS-CoV-2 vaccination. This study aimed to evaluate SARS-CoV-2 spike antibodies (anti-S Abs) following COVID-19 vaccination in CPs and healthcare workers (HCWs).

**Methods:**

We conducted a point-seroprevalence study in CPs and HCWs who received a two-dose scheme with either BNT162b2, AZD1222, or Sputnik-V vaccine. We measured anti-S Abs by quantitative immunoassay to assess humoral immune response. Besides, we quantified anti-nucleocapsid antibodies in a subgroup of individuals to determine prior infection. We compared anti-S Abs titers in both groups and stratified by vaccine type, prior infection, and clinical characteristics. We conducted a multivariate logistic regression to determine variables associated with a poor humoral response.

**Results:**

Six hundred forty-one individuals were included: 174 (27%) CPs and 467 (73%) HCWs. The median anti-S Abs titter was higher among HCWs compared to CPs (2568 U/mL vs. 1807 U/mL, p=0.002). Both CPs and HCWs with prior infection had higher anti-S Abs titter (p< 0.001). Regardless of the time since vaccination, a higher proportion of subjects with titers < 250 U/mL was observed in CPs (p< 0.001) (Fig 2). In the multivariate analysis, older age (p=0.036), AZD1222 (p=0.003), and Sputnik-V (p=0.020) were associated with lower humoral response among the entire cohort.

SARS-CoV-2 spike antibody titers among cancer patients and healthcare workers.

**Figure:**
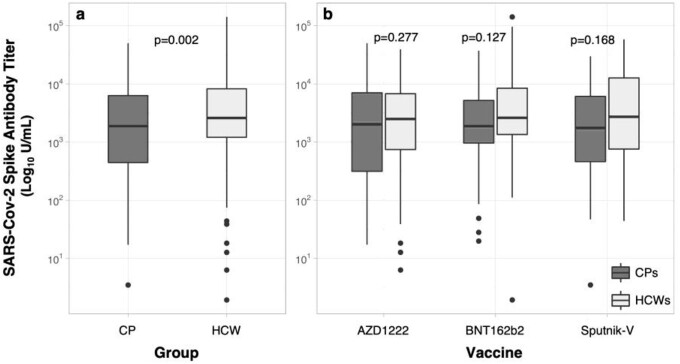

Global differences in anti-S Abs titers between CPs and HCWs groups (a) and antibody titers in CPs and HCWs groups stratified by type of received vaccine (b). Abbreviations: CP: Cancer patients, HCW: Healthcare workers.

SARS-CoV-2 spike antibody titers according to time since vaccination among cancer patients and healthcare workers.

**Figure:**
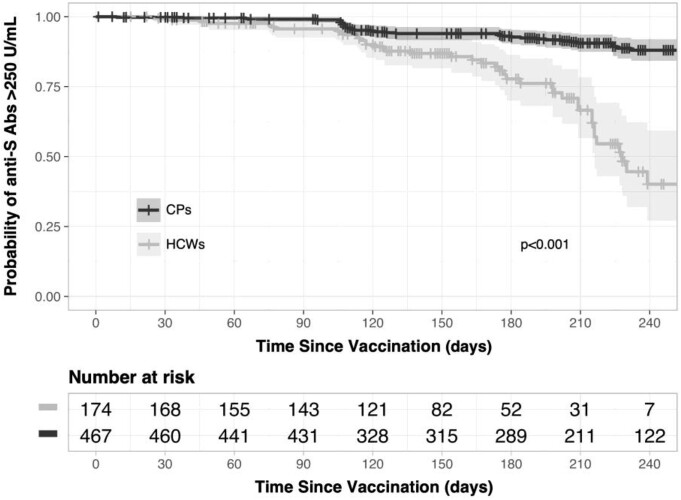

Abbreviations: CP: Cancer patients, HCW: Healthcare workers.

**Conclusion:**

In this study, both CPs and HCWs showed an adequate response to vaccination; however, CPs had lower anti-S Abs titers and a faster decline over time. Based on our results, new strategies should be assessed to sustain the humoral response to vaccination and thus decrease the COVID-19 burden among the oncologic population.

**Disclosures:**

**All Authors**: No reported disclosures.

